# Physiologically Based Pharmacokinetic Modeling in Pregnancy, during Lactation and in Neonates: Achievements, Challenges and Future Directions

**DOI:** 10.3390/pharmaceutics16040500

**Published:** 2024-04-05

**Authors:** Karel Allegaert, Sara K. Quinney, André Dallmann

**Affiliations:** 1 Clinical Pharmacology and Pharmacotherapy, Department of Pharmaceutical and Pharmacological Sciences, KU Leuven, 3000 Leuven, Belgium; 2 Department of Development and Regeneration, KU Leuven, 3000 Leuven, Belgium; 3 Department of Hospital Pharmacy, Erasmus University Medical Center, 3000 CA Rotterdam, The Netherlands; 4 Department of OB/GYN, Maternal and Pediatric Precision in Therapeutics (MPRINT) Hub, Indiana University School of Medicine, Indianapolis, IN 46202, USA; squinney@iupui.edu; 5 Bayer HealthCare SAS, Loos, France, on Behalf of Bayer AG, Pharmacometrics/Modeling and Simulation, Systems Pharmacology & Medicine–PBPK, 51368 Leverkusen, Germany; andre.dallmann@bayer.com

## 1. Introduction

**Figure 1:**
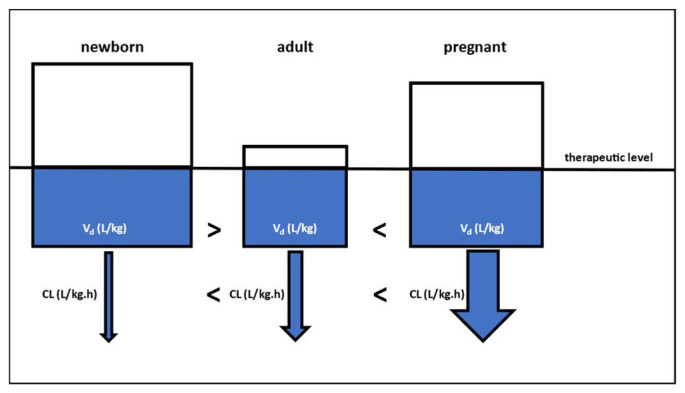
Schematic comparison of key pharmacokinetics parameters (distribution volume, Vd, and clearance, CL) between a newborn (**left**), a reference adult (**middle**) and a pregnant subject (**right**). To reach a similar therapeutic level, a proportionally higher (/kg) loading dose is needed for both newborns and pregnant subjects, compared to reference adult values. Clearance (/kg.h) is commonly lower in neonates and higher in pregnant subjects, when compared to reference adult values.

Obstetric subjects represent a special population in pharmacology. During pregnancy and in the postpartum period, various anatomical and physiological changes give rise to altered pharmacokinetics (PKs) in the mother, fetus and neonate. At the risk of oversimplification, it is expected that the body’s distribution volume (Vd, L/kg) will be higher, both during pregnancy and in infants, when compared to a reference adult population, as both the body’s water volume and fat tissue mass increase during pregnancy. Compared to the same reference adult population, the clearance (CL, L/kg.h) will commonly be higher in pregnant subjects, although some enzymes, e.g., cytochrome P450 (CYP)1A2, show the opposite effect; in infants, enzyme activity is commonly lower (Figure 1). Interestingly, these special populations usually display important time-dependent physiologies (revolving mainly around gestational age and postnatal age, respectively); therefore, these differences will also be time-dependent [1,2].

In contrast to this extensive intra- and interpatient variability, pregnant and lactating subjects and their infants are significantly underrepresented in clinical trials, leading to a dearth of in-depth information on pharmacokinetics in these populations [3]. Consequently, dosing regimens are often simply extrapolated from non-pregnant to pregnant and lactating women or allometrically scaled from adults to neonates, entailing, in both cases, considerable risks of sub-therapeutic or toxic drug effects for the mother, fetus and/or neonate [1,2,4].

In recent years, major efforts have been directed towards investigating pharmacokinetics in obstetric populations or infants, using various approaches. Population PK models enable the analysis of unbalanced or sparse observations for exploring covariates, in order to explain inter-individual variability (including pregnancy) and to adapt dosing accordingly. However, these analyses need a relevant number of observations and, owing to the non-mechanistic nature of these models, it is often not possible to disentangle the underlying physiological changes that give rise to altered drug pharmacokinetics. To mitigate this, physiologically based pharmacokinetics (PBPKs) evolved as an alternative, promising approach to predict PKs during pregnancy, during lactation and in infants.

PBPK models are mechanistic models consisting of a plethora of differential equations that deterministically predict or simulate time–concentration drug profiles within a physiologically realistic structure for the body. In such a framework, organs and tissues are compartmentalized, based on physiologic composition and size, while they are interconnected through blood flows in a parallel circuit. Hence, in contrast to population PK models, PBPK models rely on a bottom-up approach, with a priori knowledge on the physiology and anatomy of the body, as well as the physicochemical properties of the drug, integrated in a single mechanistic framework [1,5,6]. The goal of this Special Issue was to showcase strong examples of successful applications of PBPK modeling approaches in pregnant subjects, during lactation or in infants, and approaches to improve their performance.

## 2. An Overview of Published Articles

### 2.1. Pregnancy-Related Physiologically Based Pharmacokinetics Papers

Six papers focus on pregnant subjects. Le Merdy et al. described the ability to predict differences in the PKs of pregnant subjects and fetal tissue, following exposure to metoprolol, midazolam and metronidazole, using PBPK approaches (contribution 1). All these compounds undergo metabolic clearance (CYP2D6, CYP3A4 and CYP2A6). Based on assessments of available in vivo observations of pregnant and non-pregnant subjects, the authors concluded that the predictions were reasonably accurate in the maternal and fetal compartments. This was confirmed by a similar performance when compared to a previously reported effort on compounds that undergo renal clearance [7].

Two papers focus on pregnancy-related changes in phase II enzymes and drug transporters, and the plasma protein binding capacity, respectively. Using a structured PubMed assessment, Gong et al. identified information on 16 phase II drug-metabolizing enzymes and 38 drug transporters throughout pregnancy. While the retrieved information may be instrumental to further improving PBPK platforms, the sparse and conflicting evidence and knowledge gaps mainly call for additional expression profile patterns and PK data (contribution 2). As free drug concentrations are generally considered the pharmacologically active moiety, Coppola et al. explored the impact of pregnancy on the disposition of highly protein-bound drugs (e.g., tacrolimus and efavirenz). The exposure was predicted to be reduced during pregnancy, while the decrease in exposure to the total concentrations was more pronounced than the decrease in exposure to the free drug concentrations (contribution 3). Abduljalil et al. explored the applicability of PBPK modelling to predict ceftazidime pharmacokinetics during pregnancy, including in the fetal compartment (contribution 4). This drug is eliminated by the renal route, and therefore reflects the impact of pregnancy on renal clearance. While contribution 4 explored the applicability of PKPB modeling following intramuscular administration, van Hoogdalem et al. forecasted fetal buprenorphine exposure following maternal sublingual administration, using maternal–fetal PBPKs (contribution 5).

Finally, Yang et al. specifically focused on a specific disease during pregnancy (contribution 6). These authors integrated the physiological changes reported in both pregnancy and cancer populations into a PBPK modeling framework, to allow for a more accurate estimation of PK changes in pregnant subjects with cancer. They used paclitaxel and docetaxel data to develop the model, which was subsequently applied to predict acalabrutinib PKs. While docetaxel PKs were reasonably well predicted, a modified model (with a 2.5-fold increase in CYP2C8 abundance) was needed to predict paclitaxel PKs reasonably well. When subsequently using this model to predict acalabrutinib exposure, 60% lower area under the curve (AUC) and peak concentrations (C_max_ values) were predicted in pregnant subjects with cancer.

### 2.2. Neonatal Physiologically Based Pharmacokinetics Papers

Within this Special Issue, two review papers focused on PBPK modeling in the neonatal population. Dinh et al. focused on the sources of variability in the data currently available on neonatal ontogeny, while developing a neonatal PBPK model, in order to subsequently highlight data gaps where further research is needed (contribution 7). Sources of uncertainty include, among others, pathophysiology (e.g., sepsis and asphyxia), growth differences (i.e., small versus appropriate versus large for gestational age), age characteristics (e.g., gestational age and postnatal age), drug target ontogeny, hepatic and renal disposition ontogeny and protein binding (contribution 7). The data gaps highlighted by the authors concerning neonatal time-dependent physiology encompass both renal and hepatic transporter ontogeny and phase II drug metabolizing enzymes ontogeny, as well as physiological parameters like regional hepatic blood flow, small intestinal transit time and intestinal enzyme ontogeny or tissue composition. Zhang et al. focused on available information on changes in neonatal (patho)physiology as reported in published PBPK models, using a systematic search strategy (PubMed, Web of Science and Google Scholar). Based on the 56 records retained, there were only a very limited number (*n* = 3) of PBPK models with estimates related to pathophysiological changes listed (contribution 8). While sepsis, patent ductus arteriosus, acute kidney injury and asphyxia were suggested as relevant scenarios, only disease-related PBPK models on renal impairment (aminophylline) and decreased cardiac output (acetaminophen and propofol) were retrieved (contribution 8).

Finally, De Sutter et al. explored the performance of PBPK modelling on the prediction of the volume of distribution at a steady state (V_ss_) in neonates (contribution 9). Simple isometric scaling (kg^−1^) from adult V_ss_ values does not capture the developmental changes beyond body weight (like body composition, tissue composition and plasma protein pattern). The authors therefore compared the Poulin and Theil method, with Berezhovskiy correction, and the Rodgers and Rowland method, with isometric scaling, regarding V_ss_ prediction performance [8,9] (contribution 9). PBPK models were developed for 24 drugs and were compared to clinical data from 86 studies on (pre)term neonates. Isometric scaling resulted in a structured underestimation (with an average fold error of 0.61), and both corrections resulted in a lower underestimation (with an average fold error of 0.82–0.83). Compared to the isometric scaling, the Poulin and Theil method with Berezhovsky correction was superior, while the Rodgers and Rowland method was less accurate (both were based on the absolute average fold error). These findings illustrate both the applicability and the limitations of the currently available PBPK models, as well as the correction factors currently available. In a later paper, illustrations on physiological (breastfed versus formula-fed) and pathophysiological (asphyxia) characteristics that further explain the inter-individual variability in V_ss_ patterns were provided [10].

### 2.3. Lactation-Related Physiologically Based Pharmacokinetic Papers

Finally, lactation-related PBPKs merged both the maternal and neonatal PBPK models. In this Special Issue, Nauwelaerts et al. reported on a generic workflow to predict medicine concentrations in human milk (contribution 10). Following the development of PBPK models for 10 physicochemically diverse medicines in ‘non-lactating’ subjects, these models were extended to include lactation physiology, in order to explore milk/plasma ratios (based on the area under the curve) and relative infant doses. For eight medicines, the predictions were reasonable (two-fold), while overpredictions were observed for the other two medicines. This approach has potential to predict exposure with sufficient confidence to inform individual clinical decisions and perhaps also labeling decisions (contribution 10).

The relevance of PBPK modeling is further illustrated by a paper on maternal and infant tetrahydrocannabinol exposure during lactation (contribution 11). Likely related to the lipophilic characteristics of cannabinoids, there is a concern that human milk-related exposure can result in clinically relevant exposure in the infant. The authors hereby clearly illustrated that increased cannabis smoking (one to six times daily) resulted in a significant increase in the mother-to-infant plasma AUC (3.4- to 3.6-fold), with maximum infant plasma concentrations in a range between 0.084 and 0.167 ng/mL. The highest range was observed in a one-month-old infant. While these concentrations are lower than the concentration of pharmacodynamic relevance in adults, further reflection is required on how these data can guide clinical recommendations (contribution 11).

Finally, and in an effort to further inform PBPK platforms, van Neste et al. reflected on the challenges of acquiring physiological data relevant to the aims of PBPK analysis (contribution 12). Specific to lactation-related research, the authors illustrated the relevance of further improving our knowledge of the specific physiology of lactating subjects, as well as of breastfed infants. The specificities of their physiologies are illustrated by postpartum weight retention patterns, human milk intake and composition and the differences in weight gain, body composition and drug metabolism in both lactating subjects and breastfed infants (contribution 12). The authors made a call for systematic searches to ensure quality, since—following data integration as mathematical equations—this has potential to further improve postpartum, lactation and infant PBPK model performances.

## 3. Lessons Learned and Future Perspectives

PBPK models rely on a bottom-up approach, with a priori knowledge on the physiology and anatomy of the body, as well as the physicochemical properties of the drug, integrated in a single mechanistic framework [1,5,6]. Consequently, we need more structured information on (patho)physiology, preferably through the use of systematic searches. Based on data integration to mathematical equations, this can improve the performance and applicability of PBPK modeling efforts in these populations. However, these are time- and resource-consuming efforts within a multidisciplinary setting, starting with clinical researchers reporting on these physiological characteristics, followed by systematic reviews of the literature and conversion to operational PBPK models, which have to be explored for their add-on value.

Some of these (patho)physiological parameters can also be informed by integrating or scaling experimental in vitro data to the human system. For example, the expression and activity of hepatic CYP enzymes is modulated by pregnancy-related hormones (including estrogen, progesterone and cortisol), which increase progressively during pregnancy. Fashe et al. demonstrated that the exposure of sandwich-cultured human hepatocytes from adult female donors to pregnancy-related hormones, at concentrations akin to those observed during the third trimester in vivo, resulted in a 2.34 ± 0.48-fold increase in CYP3A4 protein concentrations [11]. This increase is remarkably consistent with the observed third trimester increase in the oral (unbound) clearance of midazolam [12].

Such data could help to inform or refine changes in drug metabolizing enzymes or transporter activity in pregnancy PBPK models, in case of missing or conflicting clinical PK data. Ultimately, such data could also help to align relatively diverse parameterizations of enzymatic induction in various pregnancy PBPK models. For example, in previously published pregnancy PBPK models, CYP3A4 activity in the third trimester has been shown to increase by 3.6% [13], 35% [14], 60% [15] and 100% (liver only) [16]. Similarly, CYP2C8 abundancy was kept unchanged in previously published pregnancy models, while a 2.5-fold increase in CYP2C8 abundancy—consistent with the induction observed in sandwich-cultured human hepatocytes [17]—improved the predicted AUC and C_max_ of paclitaxel in pregnant patients with cancer, as illustrated by Yang et al. (contribution 6). In these cases, in vitro data could potentially help to better address such discrepancies and could lead to a harmonization of the implemented enzymatic changes across PBPK models. This could improve the interpretability, generalizability and predictive utility of these models, which might ultimately contribute to a greater acceptance and relevance of this technology in the clinical setting.

Although physiological changes might be partially informed by in vitro experimentation, clinical data are highly valuable and required for the evaluation of PBPK models. As a critical benchmark, these data allow the refinement of model parameterizations and are essential to evaluate whether the integrated variability in various model parameters (like CYP enzyme expression) adequately translates into population variability on a PK level.

Future clinical studies are, thus, key to the advancement of PBPK modeling. In order to generate highly informative data, clinical studies should provide rich pharmacokinetic data, preferably on an individual level. In cases of studies involving pregnant individuals at delivery, the acquisition of samples from the cord blood or the placental tissue, in addition to maternal blood samples, should be considered, as these data can be highly informative for the development and evaluation of fetal PBPK models (as highlighted by Le Merdy et al., contribution 1). Similarly, if the study involves breast-feeding mothers, obtaining paired plasma and milk samples can be useful as model inputs, in the form of a milk-to-plasma concentration ratio (as pointed out by Nauwelaerts et al., contribution 10), or for evaluating the performance of the lactation PBPK model.

Echoing the remarks by Coppola et al. (contribution 3), measuring the unbound fraction of a drug in clinical studies is another relevant piece of information for PBPK models that can help to set modeling findings into the right perspective. Additionally, important co-variates such as detailed demographic information (e.g., age, gestational age, ethnicity, body weight and height) should be reported, to ensure that the simulated populations in PBPK models are true to life.

Finally, if enzymes with genetic polymorphism are involved in the metabolism of the studied drug, the genotyping of study subjects should also be considered, as this information can help to elucidate differences in enzymatic activity due to polymorphisms and contribute overall to a better understanding of inter-individual variability. Ideally, clinicians and modelers should collaborate early in the study design phase to align clinical study objectives with potential model requirements. By addressing these considerations, clinical data can be optimally leveraged in PBPK models, which, in turn, can enhance the applicability of these models in clinical decision-making and drug development.

We hope that the articles included in this Special Issue will stimulate further research in pregnant, lactating, and neonatal PBPK modeling, which we hope will ultimately contribute to a more evidence-based approach to pharmacotherapy in these vulnerable populations.
